# Surgical Implications for Nonalcoholic Steatohepatitis-Related Hepatocellular Carcinoma

**DOI:** 10.3390/cancers16162773

**Published:** 2024-08-06

**Authors:** Centura R. Anbarasu, Sophia Williams-Perez, Ernest R. Camp, Derek J. Erstad

**Affiliations:** 1 Department of Surgery, Baylor College of Medicine, Houston, TX 77030, USA; 2 Department of Surgery, Michael E. DeBakey VA Medical Center, Houston, TX 77030, USA

**Keywords:** hepatocellular carcinoma, metabolic syndrome, nonalcoholic steatohepatitis, obesity, hepatic resection

## Abstract

**Simple Summary:**

Hepatocellular carcinoma (HCC) is an aggressive form of liver cancer that arises in a background of chronic hepatic injury. Metabolic syndrome-associated fatty liver disease (MAFLD) and its severe form, nonalcoholic steatohepatitis (NASH), are increasingly common mechanisms for new HCC cases. NASH-HCC patients are frequently obese and medically complex, creating challenges when considering interventional therapies. In this review, we discuss NASH-specific challenges and the associated implications for locoregional therapies, surgical resection, and liver transplantation.

**Abstract:**

Hepatocellular carcinoma (HCC) is an aggressive form of liver cancer that arises in a background of chronic hepatic injury. Metabolic syndrome-associated fatty liver disease (MAFLD) and its severe form, nonalcoholic steatohepatitis (NASH), are increasingly common mechanisms for new HCC cases. NASH-HCC patients are frequently obese and medically complex, posing challenges for clinical management. In this review, we discuss NASH-specific challenges and the associated implications, including benefits of minimally invasive operative approaches in obese patients; the value of y90 as a locoregional therapy; and the roles of weight loss and immunotherapy in disease management. The relevant literature was identified through queries of PubMed, Google Scholar, and clinicaltrials.gov. Provider understanding of clinical nuances specific to NASH-HCC can improve treatment strategy and patient outcomes.

## 1. Introduction

Hepatocellular carcinoma (HCC) is the most common form of liver cancer, comprising 70–85% of all malignant primary liver tumors [1]. It is the sixth most common cancer worldwide, with the greatest incidence in East Asia and developing nations. HCC currently represents the third leading cause of global cancer-related death [2]. Many factors contribute to the poor prognosis associated with HCC, including a lack of effective treatment options, intrinsically aggressive tumor biology, and underlying progressive liver dysfunction. Historically, HCC arises in patients with chronic liver disease, including viral hepatitis, alcohol, and toxin exposure. More recently, the rapid global rise in metabolic syndrome-associated fatty liver disease (MAFLD) and its severe form, nonalcoholic steatohepatitis (NASH), has emerged as leading causes for HCC [3].

MAFLD affects 25% of the global population, and it is the most common cause of chronic liver disease in the United States [4]. The global prevalence for NASH is lower, approximately 5%, but the majority of metabolically driven HCC cases arise from NASH, which has a more severe inflammatory background conducive to tumorigenesis [5]. The pathogenesis of the progression of NASH to HCC is not well delineated, but present data are suggestive of multifactorial insults. Conditions characteristic of metabolic syndrome (heart disease, hypertension, obesity, dyslipidemia, and diabetes mellitus) lead to excessive lipid accumulation in the liver [6]. Hepatic steatosis causes lipotoxicity to hepatocytes, and in conjunction with increased endoplasmic reticulum and oxidative stress, this leads to hepatocellular death and immune system activation. This process, known as necro-inflammation, induces a chronic proinflammatory state, leading to hepatocellular transformation and carcinogenesis [3]. A higher proportion of patients with NASH-driven HCC (NASH-HCC) lack underlying cirrhosis compared to those with other etiologies of HCC, making early detection and surveillance more difficult in this population [7].

For patients with early HCC, the standard of care involves curative-intent surgical resection or liver transplantation. In cases for which surgical intervention is contraindicated, the use of locoregional or systemic therapies are often indicated. NASH-driven HCC is associated with multiple unique implications for clinical management. Patients with NASH are frequently obese with metabolic syndrome, features that make hepatic resection more technically challenging [8,9]. Due to these underlying medical issues, NASH patients are at greater risk for postoperative complications and have diminished capacity for recovery given their overall poor state of health [10,11]. NASH-HCC is associated with unique adverse biologic features, including increased rates of microvascular invasion (MVI) and an immunologically compromised tumor microenvironment that is less responsive to a checkpoint blockade in comparison to viral-related HCC [12,13,14,15,16]. These unique aspects must be considered with treatment selection, which is the focus of this review.

## 2. Interplay between Obesity, MAFLD/NASH, and HCC Risk

MAFLD is the hepatic manifestation of metabolic syndrome, often related to a sedentary lifestyle and the overconsumption of rapidly metabolized carbohydrates [17,18,19]. Metabolic syndrome has multiple adverse impacts on hepatic homeostatic functions, including lipid metabolism, fat storage, the enterohepatic circulation of bile salts, gluconeogenesis, and insulin sensitivity, leading to the development of MAFLD. With continued liver injury, MAFLD can progress to NASH in 20–30% of patients, resulting in a NASH prevalence of approximately 5% for the general American population [20]. NASH is associated with a risk of HCC development of approximately 2% per year [21]. While the risk mechanism for HCC tumorigenesis in the context of NASH remains poorly understood, it is thought that the NASH-induced chronic inflammatory state of hepatic parenchyma combined with metabolic dysregulation, including abnormal lipid and bile salt homeostasis, establishes an environment conducive to hepatocyte neoplastic transformation [22].

There is a significant correlation between MAFLD incidence and the presence of obesity, which has pragmatic implications for both surgical and non-surgical interventional treatments for HCC [18,23,24]. MAFLD is observed in 70% of overweight and 90% of obese patients [25]. As body mass index (BMI) increases, MAFLD essentially becomes ubiquitous and more severe disease progression correlates with a higher frequency of NASH [23,26,27]. Among the obese population, NASH prevalence is approximately 20–80%, though this can be mitigated with weight loss [28]. However, there exists a subpopulation of MAFLD patients with a normal BMI (<25), who are classified as “lean MAFLD” [29]. Certain populations, including South and East Asians, are predisposed to metabolic syndrome at lower BMIs. It is thought that such cases are partly related to genetic predisposition characterized by a higher proportion of visceral fat storage and lower metabolic tolerance for the Western lifestyle [23,30].

**Figure 1:**
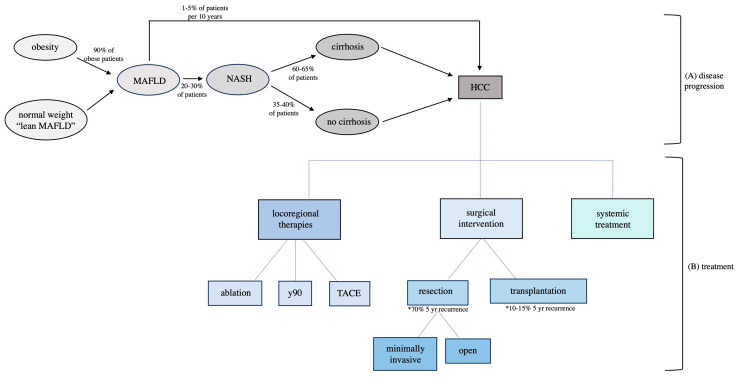
An Overview of NASH-HCC Development and Treatment Options. (**A**) The disease progression of obesity to MAFLD with the subsequent development of NASH and potential HCC. MAFLD is present in 90% of patients with obesity. Of these patients, 20–30% go on to develop NASH. Of those who develop NASH-HCC, 35–40% will not have underlying cirrhosis. (**B**) Treatment options for NASH-HCC. Locoregional therapies include ablation, y90, and TACE. Surgical interventions include resection via partial hepatectomy versus orthotopic liver transplantation. * = Resection has a higher rate of recurrence within 5 years compared to transplantation, with rates listed above. Systemic therapies include immune checkpoint inhibitors. Abbreviations: MAFLD: metabolic syndrome-associated fatty liver disease, NASH: nonalcoholic steatohepatitis, HCC: hepatocellular carcinoma, y90: yttrium 90, TACE: transarterial chemoembolization.

Both MAFLD and NASH are thought to increase the risk of HCC development, though NASH to a greater extent. In patients with MAFLD, the risk of developing HCC is approximately 1–5% over 10 years. In comparison, NASH is associated with an approximately 2–12% risk of developing HCC over 10 years [18]. Some regional variations exist, as Huang et al. have shown that the proportion of HCC cases from NAFLD is higher in the United Kingdom, India, the Middle East, and Germany. However, the HCC incidence rates from NASH are relatively similar across populations from the US, Europe, and Asia [18]. The focus of this review is on NASH-HCC, which is the more common scenario for HCC tumorigenesis and is the basis for most society guidelines (Figure 1).

## 3. HCC Screening, Diagnosis, and Implications of Obesity

Unlike most other solid organ malignancies, a diagnosis of HCC can be established by radiography alone using the liver imaging reporting and data system (LI-RADS) criteria if certain preconditions are met, including a known history of cirrhosis, hepatitis B infection, or prior HCC among adult patients. In patients with a 1 cm or larger mass on screening abdominal ultrasound and/or AFP > 20, diagnostic imaging with CT or MRI is indicated. The imaging hallmarks of HCC include non-rim arterial phase hyperenhancement, washout on portal venous or delayed phases, and capsule appearance in a mass larger than 1 cm [31]. A LI-RADS score of 5 is sufficient to establish a diagnosis of HCC. Patients with LI-RADS scores suggestive of HCC but not definitive (LI-RADS 3, 4, M, and TIV) should undergo repeat imaging at a short interval follow-up versus liver biopsy. In patients without cirrhosis, a liver biopsy is needed for diagnostic confirmation of HCC [32].

Once diagnosed, the treatment of HCC depends upon a variety of factors, including tumor characteristics (size, number of nodules, vascular invasion), the presence of cirrhosis (graded by Child–Pugh classification), and patient characteristics (performance status, comorbidities). The Barcelona Clinic Liver Cancer (BCLC) guidelines are commonly used to guide treatment selection and prognosis for patients with HCC. Patients are generally placed into one of five prognostic categories ranging from very early-stage to terminal-stage disease. Early-stage patients are favored to undergo ablation or resection, with transarterial chemoembolization (TACE) and radioembolization as backup therapies. Intermediate-stage patients are favored to undergo transplantation or TACE, and advanced-stage disease is generally limited to palliative systemic treatment [33].

Optimal patient selection for HCC screening protocols remains an ongoing challenge. Current screening guidelines for HCC involve twice yearly abdominal ultrasound examinations in patients with cirrhosis [34]. Routine surveillance in patients without cirrhosis is not currently recommended. Ultrasound has low sensitivity to detect early-stage HCC, though this can be improved with concomitant alpha-fetoprotein (AFP) measurement [35,36]. The current screening recommendations are better suited for detecting alcohol and viral-associated forms of HCC, in which the natural history of disease typically involves a period of cirrhosis before HCC occurs. Conversely, approximately 35–40% of NASH-HCC patients do not have underlying cirrhosis and therefore do not undergo routine surveillance. Additionally, among NASH patients receiving surveillance, abdominal ultrasonography is more technically challenging in the setting of obesity, which may lead to inadequate examinations and underdiagnosis. Both elevated BMI and fatty liver associated with NASH are independent predictors of poor visualization and inadequate ultrasonography due to increased hepatic, visceral, and subcutaneous adiposity [34]. The rates of inadequate ultrasonography rise proportionately with the degree of obesity: 9% inadequate ultrasound examinations in normal weight patients versus 18% in overweight patients versus 39% in morbidly obese patients [37].

Screening for NASH-HCC is further compounded by physician practice patterns. In a web-based survey of primary care physicians, it was reported that providers perceived HCC surveillance as outside their scope of practice and preferred this screening to be performed by hepatologists [35]. However, patients with metabolic syndrome and features of NASH are more likely to be diagnosed and managed by their primary care physicians, especially early in their disease states in which there are often no signs of cirrhosis to prompt evaluation by hepatology. Thus, NASH patients with moderate-to-high risk of the development of HCC may be under-surveilled. Accordingly, patients with NASH-HCC frequently present with larger tumors and an advanced tumor stage compared to patients with other underlying etiologies of liver disease [3,4,38].

## 4. Novel Approaches to NASH-HCC Risk Stratification and Earlier Diagnosis

Novel methods are needed to identify non-cirrhotic NASH patients with HCC. The American Gastroenterological Association recommends that patients with NASH, even in the absence of cirrhosis, should be stratified by non-invasive risk scores and imaging to identify high-risk patients who would benefit from HCC screening [39]. In this regard, there are multiple proposed methods for risk stratification. Serum biomarker panels, such as the GALAD scoring system, which uses gender, age, AFP, lectin-bound AFP (AFP-L3), and des-gamma carboxyprothrombin (DCP), have gained popularity as an alternative surveillance strategy that can overcome current screening limitations [34]. The GALAD protocol has been shown both in case-control and cohort studies to detect HCC with high levels of accuracy and estimated 95% specificity in patients with NASH regardless of cirrhosis [40,41].

The non-invasive assessment of liver fibrosis is another potentially effective method for the risk assessment of NASH patients. Multiple scoring systems that leverage physical and biochemical parameters have been proposed to assess fibrosis and cirrhosis, including the non-invasive fibrosis score (Fib-4), nonalcoholic fatty liver disease (NAFLD) fibrosis score (NFS), and hepamet fibrosis score (HFS). These scores are calculated by various combinations of factors, including age, sex, BMI, AST and ALT levels, platelet count, albumin level, and the presence of diabetes mellitus (DM). Among NASH patients, the NFS score accurately stratifies patients by HCC risk, while the Fib-4 score has predictive capacity for the development of both cirrhosis and HCC [42,43]. Huang et al. showed that a combination of patient risk factors, such as NASH with comorbid diabetes and obesity, as well as longitudinal assessments of fibrosis scores, such as persistently elevated Fib-4 scores or temporally increasing Fib-4 scores, was an effective approach to identify those patients as at the highest risk of developing HCC [35].

In addition to biochemical tests, imaging modalities employing magnetic resonance (MR) technology represent another non-invasive approach for risk stratifying NASH patients. Unlike ultrasonography, MR imaging captures the entire liver, is not dependent upon the operator, and is less affected by patient body habitus [44,45]. Two frequently cited MR techniques include magnetic resonance elastography (MRE) and texture analysis magnetic resonance imaging, such as proton density fat fraction (MRI-PDFF). MRE and MRI-PDFF vary in liver characteristics radiographically assessed. MRE measures hepatic tissue stiffness, a surrogate for fibrosis, whereas MRI-PDFF measures liver fat content as a means for quantifying hepatic steatosis [46,47]. MRI-PDFF has demonstrated high reliability and exam reproducibility with improved inter-rater agreement of the histologic assessment of steatosis [44]. MRE has demonstrated increased accuracy in the diagnosis and quantification of hepatic fibrosis in NASH patients regardless of patient BMI. Finally, both MRE and MRI-PDFF have shown promising results for HCC risk stratification among NASH patients, and going forward, these tests may be of value for identifying patients that could benefit from either escalated or de-escalated HCC screening [44,47,48,49,50].

Lastly, multi-omic molecular techniques are under investigation as tools for HCC risk stratification and early disease detection. Researchers have described various molecular signatures, including methylation biomarkers, metabolite profiles, circulating tumor DNA fragments, and gene expression programs that are associated with an increased risk of NASH-HCC tumorigenesis [41,51,52,53,54]. However, prospective randomized trials investigating these various risk stratification methods are necessary before their mainstream clinical application. Likewise, with the rapid application of artificial intelligence (AI) techniques in the medical field, machine learning may also provide a promising avenue for non-invasive NASH-HCC diagnosis. AI techniques have already been shown to assist MR liver imaging interpretation and can accurately diagnose liver fibrosis [55]. In other studies, machine learning has been shown to reliably predict NASH based on clinical parameters [56]. Machine learning techniques have also been used to predict HCC recurrence risk after surgical resection [57]. Going forward, the application of AI techniques for HCC detection may help lead to an earlier diagnosis, improved surveillance, and more effective patient selection for HCC screening programs.

## 5. Prevention of NASH-HCC

Prevention strategies to reduce MAFLD prevalence have the potential to greatly reduce the incidence of NASH-HCC. Currently, lifestyle changes remain the mainstay approach. Physical activity is associated with reduced free fatty acid influx to the liver, decreased oxidative stress, and improved mitochondrial function [58]. Clinically, a healthy diet and exercise can reverse signs of NASH and hepatic fibrosis, suggestive of liver recovery [28]. It has been shown that physical activity and a healthy diet reduce HCC incidence in obese patients [38]. In patients with existing HCC undergoing hepatic resection, perioperative exercise has a positive impact on recurrence-free and overall survival [59]. Therefore, the use of dietary and exercise programs prior to hepatic resection for NASH-HCC may improve outcomes in obese patients.

However, compliance remains a major challenge for behavioral modification regimens involving diet and activity. More recently, the development of novel metabolic pharmacotherapies has allowed for clinically significant medication-induced weight loss and treatment of NASH. Glucagon-like peptide-1 receptor agonists (GLP-1RA) are approved for the treatment of diabetes and off-label use as weight loss agents. Among obese patients with NASH, GLP-1RA medications have been shown to reduce hepatic inflammation and decrease the incidence of HCC [23,58]. Metformin has also been shown to decrease the risk of HCC development in patients with diabetes, and among patients with established HCC, metformin has beneficial effects on survival and recurrence rates for obese, diabetic patients [58]. Pioglitazone, a peroxisome proliferator-activated receptor gamma (PPAR-γ) agonist also used for diabetes management, is associated with a significant resolution of signs of NASH [23]. However, pioglitazone carries adverse effects, such as weight gain, peripheral edema, osteoporosis, and heart failure, and should therefore be used with caution, especially in patients with metabolic syndrome and obesity [60]. Statins, which are regularly used for the treatment of hyperlipidemia, are also associated with reduced HCC incidence and a lower risk of HCC-related mortality and recurrence [58,61]. Taken together, multiple pharmacologic agents have been shown to treat NASH and reduce the risk of HCC development, providing promise as chemoprevention agents. Some of these medications have also been shown to have beneficial impacts after the development of HCC with reduced recurrence and improved overall survival among obese and diabetic patients and those with NASH. Further investigation is necessary to determine the optimal application of these medications for chemoprevention and as HCC adjunctive treatment.

Finally, given the significant correlation between NASH and obesity, weight loss through bariatric surgery may be a reasonable option for the prevention and treatment of NASH-HCC. Bariatric surgery can induce the histologic resolution of NASH in up to 85% of patients one year after surgery [62]. In patients with MAFLD that has yet to progress to NASH, bariatric surgery induces varying rates of resolution of steatosis, inflammation, and fibrosis and confers a reduced risk of HCC development [63,64,65,66]. The different types of bariatric procedures have been evaluated across many studies, and gastric banding has been shown to be the least effective [67]. Sleeve gastrectomy is the preferred approach, as it preserves endoscopic access to the biliary tract and does not alter intestinal absorption [68,69,70]. Outcomes for the surgical resection of HCC combined with bariatric surgery have not been sufficiently studied, though the LIRESS trial from Italy is designed to evaluate the benefit of concurrent sleeve gastrectomy at the time of hepatic resection for patients with NASH-HCC [71].

There are multiple studies investigating the impact of bariatric surgery on patients undergoing liver transplantation. The sequencing of bariatric surgery and transplantation is an important factor for consideration, and there is currently no consensus. Bariatric surgery prior to transplant reduces the risk of metabolic comorbidities at the time of transplant but at the expense of increased risk of liver dysfunction. Bariatric surgery simultaneously with liver transplantation increases the operative time and risk of postoperative complications. Bariatric surgery after transplant is technically more challenging, and immunosuppression may negatively affect wound healing [63,72]. It should also be noted that bariatric surgery has limitations, as patients with Child–Pugh class B and C cirrhosis are generally not candidates for these procedures. Further investigation regarding bariatric surgery as an adjunct to surgical resection or transplantation for the treatment of NASH-HCC is needed. Finally, for these complex combined operations, the creation of multidisciplinary teams involving liver surgeons, bariatric surgeons, nutritionists, and medical oncologists to guide treatment is essential.

## 6. Impact of Obesity on Management Options for HCC

Obesity is observed in 50–80% of patients with NASH-HCC and poses unique challenges when considering interventional treatments (Table 1). With regard to surgical resection, obesity is associated with both intraoperative technical challenges and postoperative complications [24]. Patients with elevated BMI (>25) have an increased risk of mortality after hepatectomy compared to patients with normal BMI [73,74]. The risk of postoperative morbidity is also increased in obese patients undergoing major hepatic resections [75]. In a large multicenter study, BMI greater than 24 was an independent risk factor for reduced overall survival and recurrence-free survival after surgical resection for HCC [76]. In another study, it was reported that patients with metabolic syndrome and HCC had a greater risk of perioperative complications and a greater than 2-fold increased risk of death after hepatic resection [77]. Obese patients are more prone to postoperative complications, including bile leaks, intra-abdominal abscesses, pneumonia, acute renal failure, urinary tract infections, longer hospital length of stay, abdominal wall hematomas, abscesses, and dehiscence [78,79]. Obesity is also an independent risk factor for venous thromboembolism (VTE), and obese patients undergoing hepatic resection are at an increased risk of postoperative VTE compared to their normal weight counterparts [80,81,82,83,84]. Furthermore, open liver resection is associated with a higher risk of VTE than minimally invasive liver resection, thus an open liver resection for HCC in an obese individual doubles the risk of postoperative VTE-associated morbidity and mortality [85]. Patients with metabolic syndrome, regardless of BMI, have added perioperative risks, including surgical site infection, a need for blood transfusions, pulmonary complications, and myocardial infarction after liver surgery [77,86]. These risk factors should be taken into consideration by providers when counseling NASH-HCC patients regarding the risks and benefits of surgical intervention.

In addition to obesity and metabolic syndrome, cardiovascular disease and cirrhosis are also common comorbidities of NASH-HCC patients [87,88]. It is well known that patients with cardiovascular comorbidities have an increased 30-day mortality risk when undergoing major liver resections [89]. These patients also are at an increased risk of complications after locoregional therapies, such as TACE and systemic therapy, including sorafenib [90,91]. Similarly, cirrhotic patients with HCC are at an increased risk of complications from surgical interventions and locoregional therapies due to limited hepatic reserve [92,93,94]. The optimal care of NASH-HCC patients therefore includes the management of individual comorbidities to minimize adverse events from HCC-related treatments.

One method to minimize surgical risk in obese HCC patients is the utilization of minimally invasive technical approaches, such as laparoscopy and robotic surgery (Table 2). In a study by Jacoby et al., patients with metabolic syndrome who underwent robotic hepatectomy experienced no difference in operation time or estimated blood loss (EBL). Furthermore, there were no observed differences in postoperative outcomes compared to patients without metabolic syndrome [95]. Similar results were observed in a study by Sucandy et al., in which there were no significant differences in intraoperative EBL, the rate of postoperative complications, the rate of conversion to open, the need for transfusion, or the length of hospital stay among patients with elevated BMI greater than 25 compared to patients with normal BMI undergoing robotic liver resection [96]. In a study by Lin et al., the authors described shorter operative time, lower EBL, and a shorter length of stay in patients undergoing robotic hepatectomy for HCC versus open hepatectomy, and these differences were more pronounced for obese patients [97]. These findings suggest that for appropriately selected cases, the robotic approach may mitigate some of the previously identified risks of liver resections in obese patients.

Similar to robotic resection, patients who underwent laparoscopic liver resection for HCC demonstrated improved outcomes compared to patients who underwent open resection [98]. With weight-based stratification, patients undergoing laparoscopic resection showed no significant differences in operative time, complication rate, or the length of hospital stay based on their BMI [99,100]. In contrast, obesity has been associated with longer operation time and increased blood loss in open liver resections, in which the exposure can be more challenging with significant adiposity [101]. Finally, it has been shown that laparoscopic resection for HCC has similar rates of disease-free and overall survival regardless of BMI [99,102]. It should be noted that minimally invasive approaches to liver resection have limitations, particularly a learning curve that should be overcome with simpler procedures prior to attempting hepatic resections [103]. Minimally invasive hepatic resections are currently generally limited to non-anatomic partial resections or segmental anatomic resections. For most centers and providers, hepatic lobectomies or vascular reconstructions are performed with open surgery.

Locoregional therapies are essential resources for the management of HCC when curative intent treatments, including liver transplantation and surgical resection, are not options. Factors that preclude operative intervention may include HCC disease burden, liver quality, and patient comorbidities. However, increased BMI also impacts the ability to effectively administer image-guided percutaneous ablation techniques (radiofrequency and microwave). Ablative techniques have become an important tool in the clinical management of HCC, as their effectiveness has increased over time and their risk profile is reduced compared with surgery [104,105]. Ablation is generally recommended in patients with Child–Pugh class A or B disease and those with less than or equal to three nodules, each less than or equal to 3 cm in diameter per BCLC criteria [106]. Increased visceral and subcutaneous fat in obese patients can make ultrasound visualization more challenging, affecting the operator’s ability to identify and localize the tumor for treatment. It has been shown that the technical success rate of ablation in overweight patients is significantly lower than non-overweight patients, and multiple sessions are often necessary in overweight patients with HCC [106,107]. Additionally, higher amounts of visceral fat are empirically associated with a 2-fold increased risk of HCC recurrence after ablation [108]. However, overall survival remains similar post-ablation in HCC patients with MAFLD versus other etiologies of HCC [109,110].

In contrast to percutaneous ablation, TACE is performed via angiography and does not share the same technical challenges in obese patients. However, obesity is nonetheless still associated with worse outcomes in patients undergoing TACE for HCC, with higher BMI patients having significantly more residual disease, lower rates of complete response, and higher rates of progressive disease [111]. Patients with diabetes who underwent TACE were found to have an increased risk of tumor recurrence, particularly in the presence of cirrhosis [112]. Interestingly, diabetic HCC patients treated with metformin had better overall survival and progression-free survival after TACE and better overall survival and lower recurrence after radiofrequency ablation [113,114]. Even after surgical resection, the use of metformin in diabetic HCC patients has been shown to reduce the risk of postoperative recurrence [115]. The mechanism of metformin and its impact on NASH-HCC pathogenesis is not well established, but it may be beneficial to consider the use of metformin in patients with diabetes and HCC planning to undergo locoregional therapy or surgical resection.

The use of y90 is rapidly gaining popularity as another locoregional treatment modality for HCC, but less is known about its efficacy in the MAFLD-HCC population. While y90 previously was primarily used in advanced or unresectable HCC, it is now offered to patients with early HCC that are not resection candidates or as a bridge to transplantation [116]. However, y90 treatment is associated with a risk of liver-related toxicity, reflected in increased MELD scores up to two years post-treatment. It has been proposed that since MAFLD-HCC patients tend to have lower MELD scores at diagnosis, they may benefit more from y90 than patients with other etiologies of HCC [116]. It has also been shown that the metabolic comorbidities of patients with MAFLD-HCC do not impair the safety of y90 radioembolization, making it a favorable choice of locoregional treatment in this population [117]. Another potential advantage of y90 for MAFLD-HCC is its use in the neoadjuvant setting to prevent tumor progression while patients are undergoing preoperative optimization of their metabolic comorbidities prior to surgical resection or while waiting for transplantation [118]. In a randomized controlled trial, Salem et al. showed that y90 radioembolization provides significantly longer time to progression for HCC than conventional TACE [119]. In multiple studies, y90 radioembolization has been shown to be safe and effective, with comparable outcomes to other curative treatments [120,121]. Further investigation is needed regarding the impact of obesity on y90 outcomes to better understand the long-term implications of its use in the MAFLD-HCC population.

## 7. NASH-HCC Outcomes with Surgical Resection

The surgical treatment of HCC remains the cornerstone of curative therapy with overall 5-year survival rates of 70–80% [122]. NASH-HCC patients treated with surgery have comparable outcomes to patients with other etiologies of HCC, with 5-year disease-free survival rates of 60–65% [123]. A major challenge with surgical resection is the postoperative recurrence of HCC. Recurrences are classified as early (less than 2 years from resection) or late (greater than 2 years). Early recurrences are thought to be from micrometastases from the original tumor, while late recurrences are attributed to de novo tumors arising from a background field defect in the liver parenchyma [122]. Interestingly, early recurrence after surgical resection is more common among NASH-HCC patients than those with other HCC etiologies, suggesting the biology of NASH might uniquely predispose patients to micro-metastatic dissemination. In support of this argument, there is an increased prevalence of microvascular invasion (MVI) among NASH-HCC patients compared to other etiologies [13,123,124]. MVI is the microscopic observation of cancerous cells that have invaded the lumens of peritumoral hepatic vasculature [125]. It is a post hoc diagnosis that can only be made by the pathologic evaluation of surgically resected liver tissue or hepatic explants, though multiple research teams are exploring methods for preoperative MVI prediction [126,127,128,129]. Biologically, MVI implies that malignant cells are capable of infiltrating the surrounding hepatic tissue and disseminating systemically via the blood supply [130]. MVI is thus one of the most predictive pathologic biomarkers for HCC recurrence and survival [131].

Reported outcomes for the surgical resection of NASH-HCC have been inconsistent in the literature. With the higher rates of MVI noted in NASH-HCC cases, one would expect these patients to have worse overall outcomes. However, in early studies and meta-analyses, NASH-HCC patients undergoing surgical resection were shown to have comparable or better disease-free survival and overall survival to their counterparts with other etiologies of HCC [4,110,132,133,134]. This may be partly due to lower rates of cirrhosis in NASH-HCC patients, as evidenced by lower MELD scores in this population [135]. Cirrhosis is an independent risk factor for presence of MVI, and risk stratification by presence of cirrhosis has shown that disease-free survival and overall survival rates after surgical resection in cirrhotic patients is significantly lower [93,136]. In the absence of cirrhosis, patients with NASH-HCC had similar long-term outcomes to patients with non-NASH HCC [137]. Additional investigation is needed to further delineate the relationship between NASH, cirrhosis, and MVI to better understand projected outcomes for these patients.

One potential explanation for the changing trends of NASH-HCC surgical outcomes compared to viral-HCC patients is the improvement and advances in antiviral therapies. In studies reporting worse outcomes for NASH-HCC patients, the majority of viral-HCC patients had achieved viral suppression before or after surgery, leading to more favorable recurrence-free survival rates than previously reported rates in the literature [138]. Therefore, it may be more beneficial to evaluate NASH-HCC survival rates over time than in comparison to other etiologies of HCC. Overall, more recent data support the evidence that NASH-HCC is associated with earlier tumor recurrence following curative resection and shorter overall survival than other types of HCC, likely due to the increased prevalence of MVI, though the absence of cirrhosis may be a protective factor [4,138].

The use of locoregional therapies may be of significant benefit for the NASH-HCC population [131]. Researchers have described improved overall survival and disease-free survival in HCC patients with MVI who received postoperative adjuvant TACE compared to liver resection alone [139,140]. Similarly, adjuvant hepatic arterial infusion chemotherapy (HAIC) showed benefits in preventing tumor recurrence and improving survival in HCC patients with MVI [141]. In comparing various adjuvant therapies, including radiation therapy, HAIC, sorafenib, and TACE, after surgical resection, Pei et al. found that all adjuvant therapies had a positive effect compared to curative hepatectomy alone, and radiation therapy was most effective in reducing the risk of recurrence and improving overall survival [142]. More recently, a novel programmed cell death protein 1 inhibitor, sintilimab, has also preliminarily been shown to prolong recurrence-free survival following surgical resection in HCC patients with MVI [143]. Therefore, although not currently part of standard practice, there is promising value in adjuvant treatment with locoregional or systemic therapies (immunotherapy, tyrosine kinase inhibitors) for patients found to have MVI on pathologic analysis after operative resection.

Finally, a major preoperative factor to consider in determining candidacy for surgical resection in patients with hepatic malignancies is the size of the future liver remnant (FLR). For patients with background cirrhosis, an FLR of at least 40% is required to safely avoid postoperative hepatic failure. There are limited studies on the impact of BMI on liver regenerative capacity after resection. Among existing data, there is a negative correlation between BMI and regeneration. In one study, obese patients had similar regeneration rates in the first 2–3 months after major hepatic resection but slower regeneration 2–7 months postoperatively. The authors also noted a greater incidence of hepatic insufficiency after hepatectomy in the obese population [144]. In a separate, case-matched study, obese patients undergoing major hepatic resection exhibited lower postoperative liver volumetric gain rates, a marker of regenerative ability, compared to their normal weight counterparts [145]. The mechanism by which obesity influences postoperative liver regeneration is poorly understood. Some hypothesize that the degree of steatosis, proinflammatory cytokines, intraoperative ventilation requirements, and coexisting metabolic factors may all play a role [144,145]. Regardless, it is important for surgeons to consider the extent of hepatectomy needed for HCC resection in obese patients and to consider that hepatic regeneration may be marginalized in this population.

## 8. NASH-HCC and Liver Transplantation

NASH-HCC is the fastest growing indication for liver transplantation in the United States [38,146]. Transplant is the preferred method of curative therapy for HCC, as it has lower recurrence rates at 5 years (10–15%) compared to surgical resection (70%) [122,147]. Transplantation addresses both intrahepatic micrometastases (early recurrence) and the overall parenchymal defect (late recurrence), which may partly explain the difference in recurrence rates compared to surgical resection. However, limited donor organ availability can result in prolonged waitlist times and disease progression. In the United States, livers are allocated using the United Network for Organ Sharing (UNOS) system, in which patients are allocated points to create a model for end stage liver disease (MELD) score based upon their liver health and underlying diagnosis [148]. For transplant candidacy, patients must be sufficiently healthy without severe cardiopulmonary disease and no history of non-compliance or a lack of social support. Notably, BMI greater than or equal to 40 is a relative contraindication for liver transplant [149]. Given these factors, many patients with NASH-HCC, which is associated with higher BMI and lower rates of cirrhosis, do not undergo liver transplant as the primary treatment of their tumors. Instead, non-transplant interventional treatments for HCC, including surgical resection, local ablation, TACE, and y90 radioembolization, are increasingly employed in this population.

NASH-HCC patients treated with liver transplantation are at increased risk of short-term complications, but this does not appear to impact long-term survival. NASH-HCC and non-NASH-HCC patients undergoing transplantation showed no difference in recurrence-free survival outcomes [150,151]. Similarly, the overall 5-year survival rates for transplanted NASH-HCC compared to other etiologies, including viral infections and alcohol, were comparable [124]. Obesity was previously thought to be associated with increased postoperative recurrence and worse overall survival in patients undergoing transplantation [152,153]. However, this has been negated in recent studies, and obesity does not appear to influence recurrence rates, time to recurrence, or patient survival from transplant [154]. Therefore, BMI alone should not prohibit HCC patients from consideration for transplant candidacy. However, it is important to note that patients who develop HCC from NASH still carry the same risk factors to redevelop NASH post-transplantation with an estimated 5-year NASH recurrence rate of 70% [63]. Therefore, it is beneficial to also address underlying NASH in patients undergoing transplant for HCC.

## 9. Tumor Microenvironment and Medical Treatments

Compared to other solid gastrointestinal cancers, HCC is overall more responsive to immune checkpoint inhibitors (ICIs), presenting a promising therapeutic strategy. However, among HCC subtypes, NASH-HCC has been shown to be relatively unresponsive to immunotherapy compared to viral-HCC, the latter having response rates to immunotherapy of 30–40% [155]. In several studies, it was shown that the treatment of NASH-HCC with anti-PD-1 and anti-PD-L1 checkpoint inhibitors was actually associated with disease progression and reduced overall survival [3,12,16]. While the mechanism for the reduced response is not well established, it may be related to the immunosuppressed tumor microenvironment (TIME) associated with NASH-HCC. It has been demonstrated that steatohepatitis, which is central to NASH pathogenesis, reduces the effectiveness of immunotherapeutic agents by a loss of functional T-cells [156]. The NASH-HCC TIME is characterized by higher levels of M2 macrophages, myeloid derived suppressor cells, and anergic T cells, which are less responsive to a checkpoint blockade [14,16,17].

The reactivation of the immunosuppressed tumor microenvironment in NASH-HCC might represent one therapeutic strategy to increase tumor responsiveness to immunotherapy, which has been demonstrated in multiple preclinical studies (Table 3). Heinrich et al. showed that the administration of N-acetylcysteine mitigated the loss of intrahepatic T-cells and could improve the effects of various immunotherapeutic agents in slowing tumor growth [157]. In another study, Wabitsch et al. described the positive effect of metformin on improving the metabolic fitness of T-cells and restoring the sensitivity of NASH-HCC tumors to immune checkpoint inhibitors [158]. There is currently an active clinical trial examining the combination of anti-PD-1 therapy with metformin on the remodeling of the tumor microenvironment and restoration of T-cell function [159]. Zhang et al. discovered that the overexpression of Neuregulin 4, a hormonal checkpoint, can suppress T-cell exhaustion with protective effects against HCC [160].

Others have examined neutrophils in the NASH-HCC tumor immune microenvironment. Leslie et al. described targeting neutrophils in NASH-HCC tumors via a small molecule inhibitor, CXCR2, that was able to reprogram the tumor microenvironment to become more sensitized to immune checkpoint inhibition [15,16]. Zhang et al. evaluated neutrophil extracellular traps as contributors to a pro-tumorigenic environment, and their blockade was an effective suppressor of tumor growth by reducing T-cell exhaustion and expression of PD-1 and PD-L1 [161]. Finally, combination therapies have also shown preliminary promise in attenuating the immunosuppressed tumor microenvironment of NASH-HCC. The combined effect of anti-VEGF and anti-PD-L1 therapy has been shown to have a more pronounced effect on the prolongation of progression-free survival [162]. Finally, an adenosine receptor blockade has also been described as a target adjunct in improving the efficacy of existing immunotherapies, especially VEGF and PD-1 inhibitors [163,164]. While an immune checkpoint blockade is generally better tolerated than other therapies, it is also important to consider the side effect profiles of these agents. Anti-VEGF and anti-PD-1/anti-PD-L1 therapies can cause immune-related adverse events, including colitis, dermatitis, pneumonitis, and myocarditis. One must consider these potential adverse effects when combining therapies [165,166]. Taken together, strategies to re-sensitize the NASH-HCC immune ecosystem represent an innovative approach with potential to improve immunotherapy efficacy in NASH-HCC patients.

**Table 1:** Challenges and Implications of Obesity and MAFLD on Treatment of HCC.

Treatment Modality	Challenges of Obesity/MAFLD	Considerations
Transplant	- BMI > 40 relative contraindication [149] - Adiposity limits exposure/visibility in OR - Increased risk of short-term postoperative complications; comparable long-term outcomes [150,151] - High recurrence rate of NASH post-transplant [63]	- Weight loss can reduce risk of complications and recurrence of NASH [28,38,59] - Bariatric surgery (neoadjuvant or at time of transplant) can be safe/effective for weight loss and reducing metabolic comorbidities in obese/MAFLD-HCC patients [62,63,64,65,66]
Surgical Resection	- Obesity increases mortality and morbidity [73,74,75] - Increased risk of complications (cardiovascular, hepatobiliary, infectious, VTE) in obese patients undergoing hepatic resection [24,78,79] - Adiposity limits exposure/visibility in OR - Slower liver regeneration post-resection [144,145]	- Minimally invasive approach can mitigate risks of obesity on open surgery [95,96,97] - Obese patients may require larger future liver remnant - Perioperative exercise and weight loss can improve outcomes [59] - Neoadjuvant pharmacotherapies show early promise in reducing risk of HCC [139,140,141,142,143]
Locoregional Therapy	- Ablation: - ultrasound is more difficult in obese patients, lower technical success rates [106,107] - more sessions needed, increased recurrence risk after ablation in obese patients [108] - TACE: - obesity associated with more residual disease, lower complete response, and increased disease progression [111] - increased recurrence risk in obese patients with comorbid DM and/or cirrhosis [112]	- Metformin use improves survival in DM patients after ablation and TACE [113,114] - y90 is safe in patients with metabolic comorbidities, has comparable outcomes to other locoregional therapies [116,117,120,121] - Locoregional therapies can prevent tumor progression and may be useful as adjuncts to resection/transplantation while obese/MAFLD-HCC patients undergo preoperative weight loss and optimization of metabolic comorbidities [118,119]
Systemic Therapy	- MAFLD exhibits immunosuppressed tumor microenvironment [155] - MAFLD-HCC patients have lower response to PD-1/PD-L1 and immune checkpoint blockade therapies [3,12,16]	- Reactivating the tumor microenvironment in obese/MAFLD-HCC patients holds promise for increased response to systemic therapy [157,158,159,160,161,162,163,164]

MAFLD: metabolic syndrome-associated fatty liver disease, HCC: hepatocellular carcinoma, BMI: body mass index, DM: diabetes mellitus, NASH: nonalcoholic steatohepatitis, VTE: venous thromboembolism, TACE: transarterial chemoembolization, y90: yttrium-90, PD-1: programmed cell death protein 1, PD-L1: programmed death-ligand 1.

**Table 2:** Studies Evaluating Outcomes of Minimally Invasive Hepatic Resection and Impact of Obesity.

Study	Type	Study Period	Sample Size	Variable of Interest	Type of Hepatic Resection	Results
Jacoby et al. [95]	Prospective observational study	2016–2020	N = 74 *	Presence of metabolic syndrome (MS)	Robotic	No difference in operative time, EBL, conversion to open, intraoperative complications, or postoperative outcomes in patients with MS versus without MS.
Sucandy et al. [96]	Prospective observational study	2013–2017	N = 38	BMI (BMI < 25, BMI 25–35, BMI > 35)	Robotic	No difference in EBL, postoperative complication rates, rate of intraoperative conversion, need for transfusion, length of ICU stay, and length of hospital stay based on BMI.
Lin et al. [97]	Retrospective review	2010–2020	N = 208 *	BMI and surgical approach (BMI < 25, BMI > 25)	Robotic versus Open	Robotic resection associated with shorter operative time, less EBL, shorter postoperative length of stay, less risk of surgical site infection and lower rates of blood transfusion. Differences between operative time, EBL and length of stay were more significant in obese patients.
Conticchio et al. [99]	Retrospective review	2009–2019	N = 224	BMI (BMI < 30, BMI > 30)	Laparoscopic	No significant difference in operative time, complication rate, and length of stay based on BMI.
Uchida et al. [101]	Retrospective review	2010–2015	N = 68	BMI and surgical approach	Laparoscopic versus Open	Open resection associated with longer operation time and higher blood loss than laparoscopic resection. Differences were more pronounced in obese patients.
Zhao et al. [102]	Retrospective review	2003–2016	N = 201	BMI (BMI < 18.5, BMI 18.5–23, BMI > 23)	Laparoscopic	No difference in OS and RFS after laparoscopic hepatectomy based on BMI. Underweight (BMI < 18.5) associated with higher perioperative complication rates.
Xiangfei et al. [98]	Meta-analysis	1990–2017	N = 6812	Surgical approach	Laparoscopic versus Open	Laparoscopic resection associated with lower blood transfusion rate, hospital LOS, 30-day mortality rate and morbidity than open resection. Rates of 1- and 5-year OS significantly higher in the laparoscopic resection groups than open resection.

MS: metabolic syndrome, BMI: body mass index, EBL: estimated blood loss, ICU: intensive care unit, OS: overall survival, RFS: recurrence-free survival, LOS: length of stay. * = after propensity score matching.

**Table 3:** Studies Evaluating Re-sensitization of HCC Tumor Immune Microenvironment.

Author	Year	Molecule of Interest	Conclusion
Heinrich et al. [157]	2021	N-acetylcysteine	Mice with steatohepatitis given N-acetylcysteine had slowed hepatic tumor growth via restoration of CD4+ T cells and effector memory cells
Wabitsch et al. [158]	2022	Metformin	Metformin rescued efficacy of anti-PD-1 therapy against liver tumors in NASH
Zhang et al. [160]	2022	Neuregulin 4	Neuregulin 4 prevents exhaustion of cytotoxic CD8+ T cells in the liver and induction of tumor-associated macrophages
Leslie et al. [16]	2022	CXCR2 Inhibitor	Combination CXCR2 antagonist + anti-PD-1 therapy increased CD8+ T cells and tumor-associated neutrophils which switched from a protumor to anti-tumor phenotype
Zhang et al. [161]	2019	Neutrophil Extracellular Traps	Neutrophil extracellular traps blockade suppressed tumor growth by reducing PD-L1 and PD-1 expression, which are markers of T cell exhaustion
Ramadori et al. [162]	2022	Atezolizumab + Bevacizumab	Anti-PD-1 + anti-VEGF combination therapy can prolong progression free survival rates
Allard et al. [163]	2023	Adenosine A_2A_ Receptor	A_2A_ receptor functions as a tumor suppressor and restrains HCC progression by suppression of TNF-*α* secretion by macrophages
Beavis et al. [164]	2015	Adenosine A_2A_ Receptor	Efficacy of anti-PD-1 mAB can be enhanced by A_2A_ combination therapy by reversing features of CD8+ T cell exhaustion

HCC: hepatocellular carcinoma, PD-1: programmed cell death protein 1, PD-L1: programmed death-ligand 1, VEGF: vascular endothelial growth factor, TNF-α: tumor necrosis factor alpha, mAB: monoclonal antibody.

## 10. Conclusions and Future Directions

NASH is an increasingly common risk factor for HCC. Patients with NASH-HCC are often simultaneously obese with metabolic syndrome-related comorbidities, which creates a uniquely challenging clinical scenario. Curative intent treatments, including surgical resection and liver transplantation, are technically more challenging and associated with increased postoperative complications in obese patients. However, a minimally invasive approach in select patients can mitigate these risks. Locoregional therapies, especially percutaneous and ultrasound-based approaches, have limited efficacy among obese patients. Alternatively, y90 radioembolization may be a more effective approach in this population. Finally, the pathogenesis of NASH-HCC involves an immunosuppressed tumor microenvironment that is less responsive to current systemic immunotherapies. Going forward, novel agents targeting the reactivation of the tumor microenvironment as well as weight loss through lifestyle, pharmacologic, and surgical approaches may have utility in chemoprevention and as therapeutic adjuncts for the management of NASH-HCC.

## Abbreviations

AI	Artificial intelligence
AFP	Alpha-fetoprotein
AFP-L3	Lectin-bound alpha-fetoprotein
BCLC	Barcelona clinic liver cancer
BMI	Body mass index
DCP	Des-gamma carboxyprothrombin
DM	Diabetes mellitus
EBL	Estimated blood loss
Fib-4	Non-invasive fibrosis score
GLP-1RA	Glucagon-like-peptide-1 receptor agonist
HBV	Hepatitis B
HCC	Hepatocellular carcinoma
HCV	Hepatitis C
HFS	Hepamet fibrosis score
ICI	Immune checkpoint inhibitor
LI-RADS	Liver imaging reporting and data system
MAFLD	Metabolic syndrome-associated fatty liver disease
MR	Magnetic resonance
MRE	Magnetic resonance elastography
MRI-PDFF	Magnetic resonance imaging proton density fat fraction
MVI	Microvascular invasion
NASH	Nonalcoholic steatohepatitis
NASH-HCC	Nonalcoholic steatohepatitis-related hepatocellular carcinoma
NFS	NAFLD fibrosis score
PD-1	Programmed cell death protein 1
PD-L1	Programmed death-ligand 1
PPAR-γ	Peroxisome proliferator-activated receptor gamma
TACE	Transarterial chemoembolization
TIME	Tumor immune microenvironment
VEGF	Vascular endothelial growth factor
VTE	Venous thromboembolism
y90	Yttrium 90
